# Prediction of Continuous Emotional Measures through Physiological and Visual Data †

**DOI:** 10.3390/s23125613

**Published:** 2023-06-15

**Authors:** Itaf Omar Joudeh, Ana-Maria Cretu, Stéphane Bouchard, Synthia Guimond

**Affiliations:** 1 Department of Computer Science and Engineering, University of Quebec in Outaouais, Gatineau, QC J8Y 3G5, Canada; ana-maria.cretu@uqo.ca; 2 Department of Psychoeducation and Psychology, University of Quebec in Outaouais, Gatineau, QC J8X 3X7, Canada; stephane.bouchard@uqo.ca (S.B.); synthia.guimond@uqo.ca (S.G.); 3 Department of Psychiatry, The Royal’s Institute of Mental Health Research, University of Ottawa, Ottawa, ON K1N 6N5, Canada

**Keywords:** affect recognition, affective state, signal processing, image processing, face detection, machine learning, deep learning

## Abstract

The affective state of a person can be measured using arousal and valence values. In this article, we contribute to the prediction of arousal and valence values from various data sources. Our goal is to later use such predictive models to adaptively adjust virtual reality (VR) environments and help facilitate cognitive remediation exercises for users with mental health disorders, such as schizophrenia, while avoiding discouragement. Building on our previous work on physiological, electrodermal activity (EDA) and electrocardiogram (ECG) recordings, we propose improving preprocessing and adding novel feature selection and decision fusion processes. We use video recordings as an additional data source for predicting affective states. We implement an innovative solution based on a combination of machine learning models alongside a series of preprocessing steps. We test our approach on RECOLA, a publicly available dataset. The best results are obtained with a concordance correlation coefficient (CCC) of 0.996 for arousal and 0.998 for valence using physiological data. Related work in the literature reported lower CCCs on the same data modality; thus, our approach outperforms the state-of-the-art approaches for RECOLA. Our study underscores the potential of using advanced machine learning techniques with diverse data sources to enhance the personalization of VR environments.

## 1. Introduction

Affect recognition (AR) is a signal and pattern recognition process that plays a major role in affective computing [1]. Affective computing inspires the development of devices that are capable of detecting, processing, and interpreting human affective states. As such, AR is an interdisciplinary research area which includes signal processing, machine learning, psychology, and neuroscience. The affective state refers to the emotional condition or mood of an individual at a given time [1,2]. The prediction of affective states can help researchers in a variety of fields. For example, various systems can be optimized based on the affective state of the user for a better experience. AR can also aid psychologists in the diagnosis of mental disorders. AR can detect the affective state of a person by monitoring their activity and vital signs through sensors. AR can then classify affective states by analyzing physiological data, and/or visual data [3,4].

Wearable sensors are sensors that can be worn on the human body or inserted into clothing. Most state-of-the-art AR studies rely on wearable sensors for their low-cost, rich functionality, and valuable insights [1]. Integrated wearable sensor applications can be used in daily lives as they are portable and non-intrusive. They can be used to measure physiological data such as electroencephalography (EEG), electrooculography (EOG), electrocardiography (ECG), electromyography (EMG), respiratory inductive plethysmography (RIP), blood oxygen, blood pressure, photoplethysmography (PPG), temperature, electrodermal activity (EDA), inertia, position, voice, etc. Wearable-based AR systems can be used in various healthcare applications to monitor the affective states of individuals [1].

AR can also be based on visual data, which depend on multimodal features. These features are extracted from images or video. The visual features used for AR include information about facial expressions, eye gaze and blinking, pupil diameter, and hand/body gestures and poses [4]. Such features can be categorized as appearance or geometric features. Geometric features refer to the first and second derivatives of detected landmarks, the speed and direction of motion in facial expressions, as well as the head pose and eye gaze direction. Appearance features refer to the overall texture information resulting from the deformation of the neutral expression. They depend on the intensity information of an image, whereas geometrical features determine distances, deformations, curvatures, and other geometric properties [3]. There are three common data modalities currently being considered for visual AR solutions: RGB, 3D, and thermal.

Such AR solutions may be geometric-based or appearance-based. Visual AR systems that are based on images of faces usually consist of four main stages: face localization, face registration, feature extraction (predesigned or learned), and classification/regression [3]. Multimodal fusion is an additional step that is usually performed to combine multiple data modalities [3]. There are three types of multimodal fusion: early, late, and sequential fusion. Early fusion combines the modalities at the feature level, while late fusion combines the modalities at the decision level. Sequential fusion combines different modality predictions sequentially.

**Figure 1:**
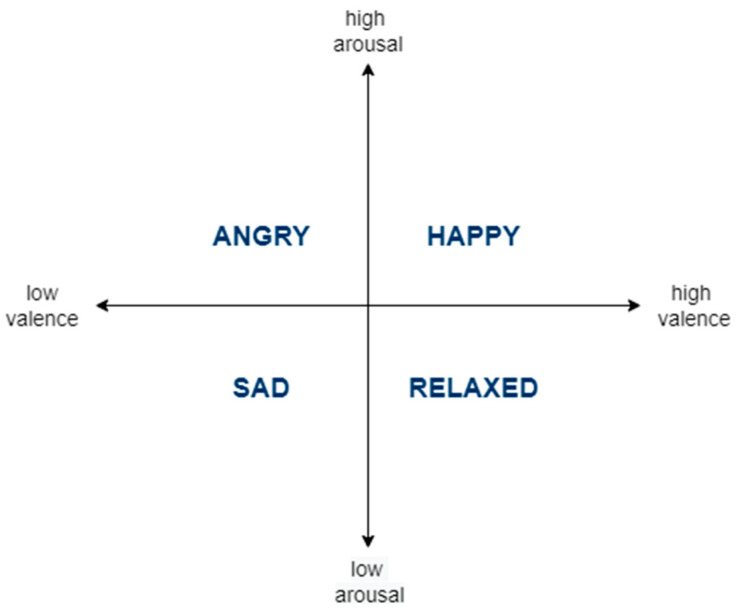
The Circumplex Model of Affective States.

The circumplex model [5] (see Figure 1) is widely used in AR studies [6,7,8,9] to predict affective states. In this dimensional model, affective states are characterized as discrete points in a two-dimensional space of valence and arousal axes. Valence is used to rate the positivity of the affective state. Arousal is used to rate the activity/energy level of the affective state. There are four quadrants in the circumplex model: low arousal/low valence, low arousal/high valence, high arousal/low valence, and high arousal/high valence. The four quadrants are attributed with the sad, relaxed, angry, and happy affective states, respectively.

In the remainder of this article, we will provide a brief background about the source of data and a literature review in Section 2. In Section 3, we will discuss our methodology and solution. We will then report our results in Section 4. Finally, we will end this article with a Conclusions section (Section 5).

## 2. Background

The application of AR requires a large amount of data, collected from a diverse group of participants. Researchers have published datasets to enable the validation and comparison of results. The data sets can consist of posed, induced, and/or natural emotions. Datasets can be grouped based on content, data modality, and/or participants [3]. Such datasets are composed of posed or spontaneous facial expressions, primary expressions or facial action units as labels, still images or video sequences (i.e., static/dynamic data), and controlled laboratory or uncontrolled non-laboratory environments. 

### 2.1. Source of Data

The remote collaborative and affective interactions (RECOLA) dataset was recorded at the University of Fribourg, Switzerland, to study socio-affective behaviors from multimodal data in the context of computer-supported collaborative work [6,10,11]. Spontaneous and naturalistic interactions were collected during the resolution of a collaborative task that was performed in pairs and remotely through a video conference. This consists of twenty-seven 5 min synchronous audio, video, ECG and EDA recordings. Even though all participants speak French fluently, they have different nationalities (i.e., French, Italian or German), which provides some diversity in the expression of emotion. The data were labelled in the arousal and valence affective dimensions, and manually annotated using a slider-based labelling tool. Each recording was annotated by six native French speakers. A combination of these individual ratings is used as ground truth label. The RECOLA data set is obtained, from [12], to assist in the analysis of continuous emotional dimensions, such as arousal and valence. The RECOLA dataset includes recordings of 27 participants, from which the recordings of 18 participants contain all types of data modalities (i.e., audio, video, ECG, and EDA). 

### 2.2. State of the Art of RECOLA for Affect Recognition

In this section, we present relevant work from the literature on the prediction of arousal and valence values from physiological, visual, and multiple sensor sources, particularly focused on the RECOLA dataset. Table 1 further reports the results from the literature we will discuss in this section. In our previous work [13], we used physiological data (EDA and ECG recordings and their features) from the RECOLA dataset to predict the arousal and valence emotional measures. The EDA and ECG signals were processed and labelled with arousal or valence annotations and a series of regressors were tested to predict arousal and valence values. The optimizable ensemble regressor achieved the best root mean squared error (RMSE), Pearson correlation coefficient (PCC), and concordance correlation coefficient (CCC). The baseline results achieved by the individual models for the gold-standard emotion sub-challenge (GES) in 2018’s audio/visual emotion challenge (AVEC) [14] are reported in terms of CCC. Their physiological results were obtained through an emotion recognition system based on support vector machines (SVMs), used as static regressors. For visual data, the authors of [14] achieved the best CCC on arousal predictions, using a multitask formulation of the Lasso algorithm. They obtained the best results on valence predictions, using an SVM. Hierarchical fusion over the different data modalities was then applied via Lasso and multitask Lasso to improve the predictions of arousal and valence values.

Amirian et al. [10] used random forests along with various schemes of fusion to predict arousal and valence values from RECOLA’s audio, video, and physiological data. Their best results were obtained by a combination of random forests and linear regression fusion over all modalities (audio, visual, and physiological). The End2You tool [15] is a toolkit for multimodal profiling that was developed by the Imperial College of London to perform continuous dimensional emotion labels of arousal and valence values. It uses raw audio, visual information (i.e., video), and physiological ECG signals as input. The authors of [15] predicted arousal and valence on RECOLA’s ECG signal and video recordings. Brady et al. [16] used RECOLA’s physiological data and baseline features, as specified in AVEC 2016 [11], to apply regression over arousal and valence values via a long short-term memory (LSTM) Recurrent Neural Network (RNN). They also extracted higher-level features from raw video and audio features using deep supervised and unsupervised learning, based on sparse coding, to ease the learning of the baseline SVM regressor. They used convolutional neural network (CNN) features to predict arousal and valence values from video recordings, using an RNN. Finally, they proposed predicting continuous emotion dimensions using a state space approach such as Kalman filters, where measurements and noise are handled as Gaussian distribution. This is to fuse the affective states (i.e., predictions) from the audio, video, and physiological data. According to [12], the results obtained by the authors of [16] are the best results obtained on RECOLA in the literature. As such, we will compare our results to theirs.

Han et al. [17] used RECOLA’s visual features to predict arousal and valence values through an RNN. Weber et al. [18] used visual features provided by RECOLA’s team in 2016 to perform regression via a SVM with late subject, multimodal fusion (at decision/prediction-level). The authors of [19] exploited CNN features from RECOLA’s videos as well as an RNN to estimate valence values. CNNs have also shown promising results when used to perform AR. AlexNet [7] was used in a number of studies to obtain deep visual features. AlexNet has also been applied on emotion recognition, where results demonstrated evident performance enhancements [8,9]. In this work, we exploit CNNs, such as ResNet and MobileNet, to predict continuous dimensional emotion annotations in terms of arousal and valence values from visual data.

Povolny et al. [20] presented a multimodal emotion detection algorithm using audio, bottleneck, and text-based features as well as the features suggested by Velstar et al. [11]. The set of visual features in [11] were accompanied with CNN features, extracted from hidden layers, after training the CNN for landmark localization. The authors of [20] proposed multiple linear regression systems, trained on individual feature sets, for predicting the arousal and valence emotional dimensions. In comparison to [20], Somandepalli et al. [21] used Kalman filters for decision level fusion. They first used support vector regression (SVR) to perform predictions from unimodal features, where predictions are noisy estimates of arousal and valence. The output of the SVR models was inputted to the Kalman filters for fusion. They later [22] proposed facial posture cues and a voicing probability scheme to deal with the multimodal nature of the problem. Table 1 summarizes the results of the above-mentioned state-of-the-art studies and further compares them to our results, as they will be presented in the remainder of the paper. A lower RMSE shows better performance. On the other hand, higher PCC and CCC show better performance.

**Table 1:** Summary of results from the literature on prediction of arousal and valence values.

Data Type	Prediction	Reference	Technique	Results (RMSE, PCC, CCC)
Physiological	Arousal	Current	ECG + EDA Optimizable Ensemble	0.0168, 0.9965, 0.9959
		[13]	ECG + EDA Optimizable Ensemble	0.0154, 0.9976, 0.9967
		[10]	ECG Random Forests	N/A, N/A, 0.097
		[10]	EDA Random Forests	N/A, N/A, 0.074
		[14]	ECG SVM	N/A, N/A, 0.065
		[14]	EDA SVM	N/A, N/A, 0.029
		[15]	ECG End2You	N/A, N/A, 0.154
		[16]	ECG RNN	0.218, 0.407, 0.357
		[16]	EDA RNN	0.250, 0.089, 0.082
	Valence	Current	ECG + EDA Optimizable Ensemble	0.0083, 0.9985, 0.9978
		[13]	ECG + EDA Optimizable Ensemble	0.0139, 0.9954, 0.9946
		[10]	ECG Random Forests	N/A, N/A, 0.139
		[10]	EDA Random Forests	N/A, N/A, 0.206
		[14]	ECG SVM	N/A, N/A, 0.043
		[14]	EDA SVM	N/A, N/A, 0.058
		[15]	ECG End2You	N/A, N/A, 0.052
		[16]	ECG RNN	0.117, 0.412, 0.364
		[16]	EDA RNN	0.124, 0.267, 0.177
Visual	Arousal	Current	MobileNet-v2 CNN	0.1220, 0.7838, 0.7770
		[10]	Random Forests	N/A, N/A, 0.514
		[14]	Multitask Lasso	N/A, N/A, 0.312
		[15]	End2You	N/A, N/A, 0.358
		[16]	CNN + RNN	0.201, 0.415, 0.346
		[17]	RNN	N/A, N/A, 0.413
		[18]	SVM + Subject Fusion	N/A, N/A, 0.682
	Valence	Current	MobileNet-v2 CNN	0.0823, 0.7789, 0.7715
		[10]	Random Forests	N/A, N/A, 0.498
		[14]	SVM	N/A, N/A, 0.438
		[15]	End2You	N/A, N/A, 0.561
		[16]	CNN + RNN	0.107, 0.549, 0.511
		[17]	RNN	N/A, N/A, 0.527
		[18]	SVM + Subject Fusion	N/A, N/A, 0.468
		[19]	CNN + RNN	0.107, 0.554, 0.507
Multimodal	Arousal	Current	Optimizable Ensemble + MobileNet-v2	0.0640, 0.9435, 0.9363
		[10]	Random Forests + Linear Regression	0.118, 0.776, 0.762
		[14]	Hierarchical Fusion + Lasso	N/A, N/A, 0.657
		[16]	Kalman Filters	0.115, 0.774, 0.770
		[20]	Multiple Linear Regressors	N/A, N/A, 0.833
		[22]	SVR + Kalman Filters	N/A, N/A, 0.703
	Valence	Current	Optimizable Ensemble + MobileNet-v2	0.0431, 0.9454, 0.9364
		[10]	Random Forests + Linear Regression	0.104, 0.634, 0.624
		[14]	Hierarchical Fusion + Multitask Lasso	N/A, N/A, 0.515
		[16]	Kalman Filters	0.100, 0.689, 0.687
		[20]	Multiple Linear Regressors	N/A, N/A, 0.596
		[22]	SVR + Kalman Filters	N/A, N/A, 0.681

N/A (not applicable); RMSE (root mean squared error); PCC (Pearson correlation coefficient); CCC (concordance correlation coefficient); ECG (electrocardiogram); EDA (electrodermal activity); SVM (support vector machine); RNN (recurrent neural network); CNN (convolutional neural network); SVR (support vector regression).

The following references showed the potential of using RNNs and CNNs to perform AR. Gunes and Schuller [23] trained two separate deep CNNs. The CNNs were pre-trained on a large dataset, and then fine-tuned on a dataset of audio and video. Gunes et al. [24] showed the potential of using LSTMs for dimensional emotion prediction. Ringeval et al. [25] used a LSTM RNN to perform regression for dimensional emotional recognition based on visual, audio, and physiological modalities. Chen et al. [26] used LSTMs to identify the long-term inter-dependency within segments of a multimedia signal. They proposed a new conditional attention fusion scheme, in which modalities are weighted according to their current and previous features. Tzirakis et al. [27] used a shallow network followed by identity mapping to extract features from raw audio and video signals. The obtained features were then inputted into a two-layer LSTM. The LSTM was trained from end-to-end instead of training it on individual components separately. This approach outperformed traditional approaches based on baseline handcrafted features in the RECOLA dataset. Huang et al. [28] applied a deep neural network and hypergraphs for emotion recognition using facial features. Facial features were extracted from the last fully connected layer of the trained CNN, which were then treated as attributes for the hypergraph. Ebrahimi et al. [29] used a CNN to extract features that were input into an RNN. The RNN categorizes the emotions in RECOLA’s video recordings.

Most state-of-the-art studies performed complex processing, feature extraction, and multimodal fusion processes. Despite these efforts, the prediction performances of their models can still be improved. We were able to perform simple processing and achieved better results using only the EDA and ECG recordings of RECOLA in [13]. In this study, we aim to further improve our prediction performance and initiate our work on the video recordings of RECOLA.

### 2.3. Study Contributions

**Figure 2:**
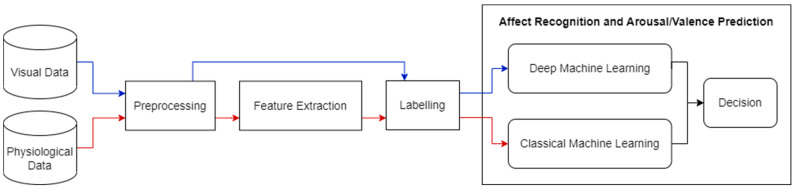
An overview of our proposed solution. The red line denotes physiological data, and the blue line denotes visual data.

Our goal in this work is to design and develop a novel adaptable intervention to remediate cognitive impairments in people with schizophrenia using virtual reality (VR), based on synergistic computer science and psychology approaches. A novel machine learning approach which depends on visual and physiological sensory data is required to adaptively adjust the virtual environment to the affective states of users. In the future, we also aim to automatically optimize the level of cognitive effort requested by users, while avoiding discouragement. AR can help determine the affective states of users and this study presents the first milestone of this project. In our proposed solution, a multi-sensory system will be used to improve the prediction of affective states. The information from the various data sources in the system will be treated to predict the affective state of the user, during VR immersion, through classical and deep machine learning techniques. Figure 2 displays a high-level diagram of our proposed solution. It is composed of subsystems: one system for each data modality, namely the visual (video) and physiological (EDA and ECG) data modalities. We chose to focus on one data modality at a time to perfect the results for each modality first, before we combine all modalities in our final system (i.e., multimodal fusion). In [13], we operated on physiological data from RECOLA’s physiological signal recordings of EDA and ECG. We processed the EDA and ECG signals by applying time delay, early features fusion, arousal and valence annotation labelling, and data shuffling and splitting. We used early fusion to combine the EDA and ECG modalities at the feature level. We exploited an optimizable ensemble regressor for the purpose of predicting continuous dimensional emotion annotations in terms of arousal and valence values.

In this study, we extend our previous work from [13] by (1) adding preprocessing operations; (2) applying feature standardization; (3) applying feature selection; (4) testing additional regressors, namely tree regressors and exploring RNNs, specifically a bidirectional LSTM (BiLSTM); and (5) introducing decision fusion. Furthermore, we introduce an additional source of data, namely video recordings. We initially use data from RECOLA as a proof-of-concept mechanism. In the future, we will operate on real data that we will collect in our laboratory.

## 3. Materials and Methods

As we will further present in this section, we processed the physiological signals and visual recordings and labelled them. Then, we performed continuous dimensional emotion predictions of arousal and valence values, through classical machine learning and deep learning techniques. Specifically, we experimented with tree and ensemble regression as well as a BiLSTM RNN for physiological data. We chose to work with tree and ensemble models because they are known to be fast and effective [30]. On the other hand, we chose to work with a BiLSTM RNN because it is capable of processing data in the form of time series and sequences, which suits our needs. BiLSTM RNNs can learn from past and future samples/time steps. For visual data, we experimented with ResNet and MobileNet CNNs as they offer a good trade-off between accuracy, speed, and size.

### 3.1. Processing of Physiological Signals

**Figure 3:**
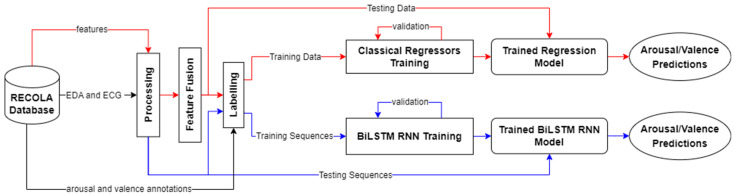
An overview of our approach for physiological data. For physiological data, we experimented with classical regressors as well as a BiLSTM RNN to predict arousal and valence values. The red lines represent the flow for the classical regression approach and the blue lines represent the flow for the BiLSTM RNN approach. The black lines represent mutual steps between the two approaches. Note that the predictions from the two approaches are separate and are not combined.

In our work, RECOLA recordings of 18 participants were used for training and validation, as well as testing purposes. All records were preprocessed by applying time delay and sequencing, early feature fusion, replacement of missing values, feature standardization/normalization, arousal and valence annotation labelling, and data shuffling and splitting. After processing the physiological data, classical regressors and a BiLSTM RNN were used for predicting arousal and valence values. Figure 3 details our approach for processing physiological data.

RECOLA’s physiological recordings (i.e., EDA and ECG) were sampled at a rate of 1000 samples per second. This means that one sample was captured every 1 millisecond. On the other hand, RECOLA’s audio and video recordings were sampled every 40 ms. Similarly, the physiological features were calculated every 40 ms as well. To enable the synchronous use of data, we subsampled the EDA and ECG signals by considering only the readings occurring every 40 ms. However, the corresponding EDA and ECG features in RECOLA were only calculated after 2 s of recording. Therefore, we skipped the readings that were collected before that time. As a result, the first 50 samples of the recordings were discarded.

We used the baseline EDA and ECG features of RECOLA, as described in [11]. Skin conductance response (SCR) is EDA’s rapid, transient response, whereas skin conductance level (SCL) is EDA’s slower, basal drift. In [11], both SCR and SCL are extracted from the EDA signal through a third-order Butterworth filter, at different cut-off frequencies. In addition to the EDA readings, there are 62 EDA features, which include: the slope, the fast Fourier transform (FFT) entropy and mean frequency of the SCR, the mean, its first-order derivative, and the negative part of its derivative for EDA, SCR, and SCL; the standard deviation, kurtosis, and skewness of EDA, SCR, and SCL; the proportion of EDA, SCR, and SCL; the x-bound of EDA, SCR, and SCL; the non-stationary index (NSI) and normalized length density (NLD) of EDA, SCR, and SCL; and deltas of all of the above. Besides the ECG readings, there are 54 ECG features, which consist of the heart rate and heart rate variability; the zero-crossing rate; the first 12 FFTs; the entropy, mean frequency, and slope of ECG’s FFT; the first four statistical moments (mean, standard deviation, kurtosis, and skewness); NSI and NLD; the power at very low, low, high, and low/high frequencies; and deltas of all of the above. We fused the EDA and ECG features together into one matrix. The resulting matrix was 132,751 samples (rows) × 118 features (columns) (1 EDA reading + 1 ECG reading + 62 EDA features + 54 ECG features) in size.

During our work, we observed that some of RECOLA’s physiological feature vectors contained missing values. A missing value can also be called not a number (NaN), which represents an undefined or unrepresentable value; for example, the result of dividing a number by zero. Missing values can negatively influence the performance of machine learning techniques. Hence, we replaced them with zeros.

To study the impact on the regression performance, multiple data preprocessing techniques were investigated. In particular, we explored standardizing/normalizing the EDA and ECG features using the z-score method:

(1) $$x_{i}’=z_{i}=\frac{x_{i}-\mu }{\sigma }$$

where $x_{i}$ is a sample feature value; while 𝜇 and 𝜎 are the mean and the standard deviation of the feature vector, respectively. This approach standardized the data in each feature vector to have a mean of 0 and a standard deviation of 1.

In this study, the main sources of outliers in the features could be due to sensor malfunctions, human errors, noise from participant-specific behaviors, or natural variations between participants. We implemented a feature selection mechanism which depends on the number of outliers in a given feature vector. To be considered an outlier, the value of a feature should be “more than three scaled median absolute deviations from the median” [30]. Our method first detects outliers within each feature vector. Then, it checks if the number of outliers in any feature vector is higher than an acceptable percentage of the data. If so, the corresponding feature vector is discarded. Only feature vectors that contain an number of outliers that is less than or equal to the acceptable percentage of the data are selected for machine learning. For instance, when we set the acceptable percentage of outliers to 5%, 50 features are selected as a result. When we set the acceptable percentage of outliers to 15%, 103 features are selected as a result. The more outliers are allowed, the more features pass the features selection test.

Data shuffling is necessary to ensure the randomization and diversity of the data. The data were shuffled and split, where 80% were used for training and validation, and 20% were used for testing. The shuffled indexes were saved for use at later stages of our work. Since recordings were for different participants and time steps, we needed the shuffled indexes to ensure that the training, validation, and testing records were matching for all data modalities. Table 2 represents the breakdown of the data.

For annotation labelling, the first 50 annotations were ignored to match the physiological recordings. The remaining annotations were accordingly used to label the corresponding physiological samples. All labelling and fusion of data samples and features were carried out based on the recording time.

### 3.2. Processing of Visual Data

**Figure 4:**
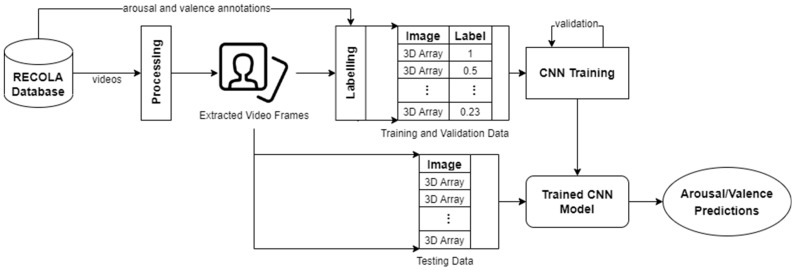
An overview of our approach for visual data.

In our work, 18 RECOLA videos were preprocessed. All videos were preprocessed by applying frame extraction and sequencing, face detection and cropping, annotation labelling, and data augmentation. After processing, the extracted images (i.e., video frames) of participants’ whole faces were inputted into CNNs for predicting arousal and valence values. Figure 4 illustrates our approach for processing visual data.

The videos available in the RECOLA dataset are approximately 5 min long each. They were processed by extracting their video frames at a rate of 25 frames per second. As a result, we obtained an image frame every 40 ms of video recording. That is a total of approximately 7500 frames per video. To match the physiological data, we skipped the first 50 frames again and ensured that the data were synchronous (i.e., had all samples for the same times of recording). For example, we would have EDA and ECG readings, EDA and ECG features, and a video frame collected at the 40th millisecond of recording, the 80th millisecond of recording, and so on. The same shuffling indexes we used for shuffling the physiological data were used here to randomize and shuffle the video frames to ensure data coherence. Visual data were then split using an 80–20% split, as in the case of physiological data. Table 3 represents the breakdown of the visual data.

Face detection was then applied to narrow the prediction area. We used the cascade object detector based on the Viola–Jones algorithm to detect people’s faces [31]. Following face detection, we noticed that the algorithm failed to detect faces in some of the obtained video frames. Hence, we cropped these images according to the face coordinates of the nearest image with a detected face. In the best-case scenario, the nearest image with a detected face would be the image preceding or following the image with a missed face. In the worst-case scenario, the algorithm would have failed to detect faces in a group of images, where the nearest image with a detected face would be more than one video frame away. In this case, the coordinates of the face might be off due to the movement of the participant in the video. Thus, manual intervention to edit the images was required.

Similar to the approach in Section 3.1, the first 50 annotations (2 s × 25 samples per second) were ignored. The remaining annotations were accordingly used to label the corresponding visual samples. All labelling and fusion of data samples and features were carried out based on the recordings time.

### 3.3. Machine Learning

We exploited both classical and deep machine learning methods to perform continuous dimensional emotion predictions of arousal and valence values.

#### 3.3.1. Classical Regression

We trained and validated four tree regressors (fine, medium, coarse, and optimizable tree), as well as ensembled regressors such as boosted trees, bagged trees, and an optimizable ensemble. A fine regression tree is small and has a minimum leaf size of 4 [30]. A medium regression tree has a minimum leaf size of 12. A coarse regression tree is larger, with a minimum leaf size of 36. An optimizable regression tree optimizes training hyperparameters (i.e., minimum leaf size) using a Bayesian optimizer. Boosted trees represent an ensemble of regression trees using the LSBoost algorithm. Bagged trees represent a bootstrap-aggregated ensemble of regression trees. An optimizable regression ensemble optimizes training hyperparameters (ensemble method, number of learners, learning rate, minimum leaf size, and number of predictors to sample) using Bayesian optimization.

We performed 5-fold cross-validation during training to protect against overfitting. For the classical machine learning approach, we used RECOLA’s EDA and ECG recordings along with their features as described earlier. Table 4 displays the dimensions of the datasets we used for classical regression. As discussed in Section 3.1, we fused the baseline EDA and ECG features of RECOLA, described in [11].

#### 3.3.2. Recurrent Neural Network (RNN) Regression

We also trained and validated a BiLSTM RNN, using sequences of ECG and EDA, to predict arousal and valence values. A BiLSTM network learns bidirectional long-term dependencies between time steps of time series or sequence data [30]. These dependencies can be useful when we want the network to learn from the complete time series at each time step. In this case, the EDA and ECG signals were processed into sequences. Since the physiological signals contained in RECOLA were sampled every 1 millisecond, unlike the other modalities, which were sampled every 40 ms, the physiological signal samples were grouped into 40 ms sequences. As a result, we had 132,751 labelled sequences of EDA and ECG readings (without features) that were composed of 40 samples each. The data sequences were divided into a training set of 106,201 sequences and a testing set of 26,550 sequences. The training set was further broken with an 80–20% split to allow for validation. As a result, the final training set contained 84,960 sequences, and the validation set contained 21,241 sequences. We did not use the feature sets for training the BiLSTM RNN model, since it is a deep learning approach, where the neural network learns features directly from the data. Thus, the processing steps related to physiological features from Section 3.1 were not applied on the data used for RNN regression.

Table 5 shows the training parameters of the BiLSTM network. We experimentally set the initial learning rate to 0.0001, and the number of epochs to 30. As there were 84,960 training sequences, we set the minimum batch size to 9 in order to evenly divide the training data into 9440 equal batches and ensure the whole training set was used during each epoch. This resulted in 9440 iterations per epoch (84,960/9 = 9440). For validation frequency, we divided the number of iterations by 2 to ensure that the training process was validated at least twice per training epoch. We set the number of hidden units to 125. Since the network was bidirectional, the number of hidden units multiplied by 2; that is 250 units. The hidden state can contain information from all previous samples/time steps, irrespective of sequence length. A BiLSTM can include past and future information. Lastly, we used the stochastic gradient descent with momentum (SGDM) optimizer for training.

#### 3.3.3. Convolutional Neural Network (CNN) Regression

We also experimented with two pretrained MATLAB CNNs: ResNet-18 and MobileNet-v2. ResNet-18 is a CNN that is 18-layers-deep, whereas MobileNet-v2 has a depth of 53 layers [30]. Both of these pretrained CNNs are capable of classifying images of 1000 object categories, such as a keyboard, mouse, pencil, and many animals. As a result, these networks have learned rich feature representations for a wide range of images. ResNes-18 and MobileNet-v2 have an image input size of 224 × 224 × 3.

To fine-tune the pretrained CNNs for regression to predict arousal and valence values, we customized the layers of each CNN to suit our needs and apply data augmentation. We, thus, replaced the image input layer to make it accept images of size 280 × 280 × 3. Additionally, we replaced the final fully connected layer and the classification output layer with a fully connected layer of size 1 (the number of responses, i.e., arousal/valence value) and a regression layer. The convolutional layers of the CNNs extract image features that are then used by the last learnable layer and the final classification layer to classify the input image [30]. These layers have information about converting the extracted features into class probabilities, loss values, and predicted labels. In the cases of ResNet-18 and MobileNet-v2, the last learnable layer is the fully connected layer. We adjusted the learning rates of the last learnable layer in order to make the CNNs learn faster in the new fully connected layer than in the transferred/pretrained convolutional layers by setting the learning rate factors for weights and biases to 10.

The amount of training data was increased by applying randomized data augmentation. Data augmentation allows CNNs to train to be invariant to distortions in image data and helps to prevent overfitting, by preventing the CNN from memorizing the exact characteristics of training images. We use augmentation options such as random reflection in the *x*-axis, random rotation, and random rescaling. As aforementioned, we replaced the image input layer of the pretrained CNNs (ResNet-18 and MobileNet-v2) to allow them to take larger input images of size 280 × 280 × 3, but the images in our video frames did not all have this size. Therefore, we used an augmented image datastore to automatically resize the images. We also specified additional augmentation operations to perform on the images in order to prevent the CNNs from memorizing image features. We randomly reflected the images along the vertical *x*-axis, and randomly rotated them from the range [–90, 90] degrees, and randomly rescale them from the range [1, 2]. These changes do not affect the contents of the training images; however, they will help the CNNs in extracting/learning more features from the images.

We modified the training options and parameters depending on the size of our input data. Table 6 summarizes the training parameters we used for training the CNNs. We experimentally set the initial learning rate to 0.0001, and the number of epochs to 30. As there were 84,960 training images, we set the minimum batch size to 9 in order to evenly divide the training data into 9440 equal batches and ensure the whole training set was used during each epoch. This resulted in 9440 iterations per epoch (84,960/9 = 9440). For validation frequency, we divided the number of iterations by 2 to ensure that the training process was validated at least twice per training epoch. We used the SGDM optimizer for training.

#### 3.3.4. Decision Fusion

We fused the testing predictions from various regressors and neural networks by averaging them to observe how this fusion affected the prediction performance. Let N be the number of trained regressors and neural networks, and P be the predictions set obtained by model i; the final predictions set, $P_{final}$, can then be computed as follows:

(2) $$P_{final}=\frac{P_{1}+P_{2}+\ldots +P_{n}}{N}=\frac{\sum_{i=1}^{n}P_{i}}{N}$$

## 4. Results and Discussion

After training the aforementioned models, we tested them by predicting the arousal and valence values on the testing sets. This helped to evaluate the performance of the trained models when they were presented with new data. We used the RMSE, PCC, and CCC performance measures to enable a comparison with similar work in the literature. The RMSE simply measures the root of the mean of squared difference between the set of arousal/valence predictions and the set of the actual values [11,23,30]. The RMSE is calculated as follows:

(3) $$RMSE=\sqrt{\frac{\sum_{i=1}^{n}{\left({\hat{y}}_{i}-y_{i}\right)}^{2}}{N}}$$

PCC measures the linear correlation between the set of arousal/valence predictions and the set of the actual values [11,23,30]. PCC is estimated using the following formula:

(4) $$PCC=\rho =\frac{\sum_{i=1}^{n}\left({\hat{y}}_{i}-\mu_{\hat{y}})(y_{i}-\mu_{y}\right)}{\sqrt{\sum_{i=1}^{n}{\left({\hat{y}}_{i}-\mu_{\hat{y}}\right)}^{2}\cdot \sum_{i=1}^{n}{\left(y_{i}-\mu_{y}\right)}^{2}}}$$

CCC measure combines PCC with the square difference between the mean of the set of arousal/valence predictions and the set of the actual values [11,30]. The CCC can be evaluated by the following equation:

(5) $$CCC=\rho_{c}=\frac{2\rho \sigma_{\hat{y}}\sigma_{y}}{{\sigma_{\hat{y}}}^{2}+{\sigma_{y}}^{2}+{\left(\mu_{\hat{y}}-\mu_{y}\right)}^{2}}$$

where N is the total number of samples; ${\hat{y}}_{i}$ and $y_{i}$ are the predicted and actual values for a given sample at time/index, i; ρ is PCC between the predicted and actual sets; $\sigma_{\hat{y}}$ and $\sigma_{y}$ are the standard deviations of the predicted and actual sets; and $\mu_{\hat{y}}$ and $\mu_{y}$ are the mean values of the predicted and actual sets. A smaller RMSE value represents better performance. On the other hand, larger PCC and CCC values represent better performance. The following sections will discuss our results and prediction performance in further detail.

### 4.1. Results on Physiological Data

This section contains a discussion of the results we obtained on physiological data from RECOLA’s database.

#### 4.1.1. Classical Regression Results

As described in Section 3.3.1, we tested seven regression models including four tree regressors and three ensemble models. Our results for the three ensemble models were similar to our previous results from [13], where the optimizable ensemble regressor outperformed the other models. For arousal predictions, it achieved a testing RMSE, PCC, and CCC of 0.0173, 0.9966, and 0.9956, respectively. For valence predictions, it achieved a testing RMSE, PCC, and CCC of 0.0126, 0.9957, and 0.9950, respectively. As far as we know, these results are better than any results reported in other state-of-the-art studies using the physiological data from RECOLA.

Replacing missing values with zeros and feature standardization/normalization through the z-score method positively influenced the prediction performance of both arousal and valence predictions. The best performance was achieved via the optimizable ensemble regressor. For arousal, it achieved a testing RMSE, PCC, and CCC of 0.0170, 0.9967, and 0.9958, respectively. For valence, it achieved a testing RMSE, PCC, and CCC of 0.0106, 0.9971, and 0.9965, respectively. As such, we can conclude that a processing mechanism which includes replacing missing values and z-score standardization improves the prediction performance when predicting the emotional measures of arousal and valence.

After feature selection, our prediction performance improved. A feature selection mechanism which allows only 5% of outliers (50 features) produced better results for arousal predictions. On the other hand, allowing up to 15% of outliers (103 features) produced better results for valence predictions. With 50 features selected, the optimizable ensemble regressor achieved an RMSE, PCC, and CCC of 0.0168, 0.9965, and 0.9959 when predicting arousal values. With 103 features selected, the optimizable ensemble regressor achieved an RMSE, PCC, and CCC of 0.0083, 0.9985, and 0.9978 when predicting valence values.

**Figure 5:**
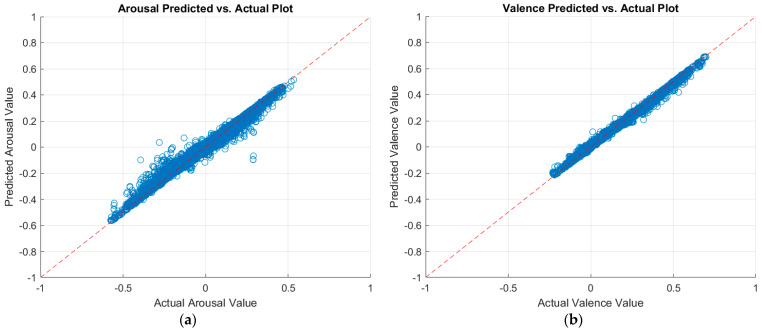
Plots of the predicted versus actual values of (**a**) arousal and (**b**) valence as per the optimizable ensemble model.

Table 7 summarizes the validation and testing results of the classical machine learning regressors. The validation results were computed through 5-fold cross-validation over the training data. The testing results were obtained by having the trained model predict arousal and valence values on the testing set. The row corresponding to the highest prediction performance, for each case, is displayed in bold font in the tables. Figure 5 displays a plot of the predicted arousal and valence values against the actual values, as per the best model. A perfect regression model has predicted values equal to the actual values; in which case, all points would be on the diagonal line [30]. The vertical distance from the diagonal line to any point represents the prediction error for that point. Good models would have small errors, where the predictions are scattered near the line.

#### 4.1.2. Recurrent Neural Network (RNN) Results

We tested the trained BiLSTM RNN with a dataset of 26,550 sequences. Each sequence was 40 ms/samples in length. Table 8 summarizes the validation and testing results from BiLSTM regression in terms of the RMSE, PCC, and CCC performance measures.

This shows that the classical regression results are better than the BiLSTM results when predicting arousal and valence values. In spite of this observation, it is worth noting that our results are comparable to the results obtained in [16] and outperform the baseline results from [14]. For arousal, our results are better than those of [16], but that is not the case for valence.

### 4.2. Results on Visual Data

As described in Section 3.3.3, we tested two CNN models: ResNet-18 and MobileNet-v2. Table 9 summarizes the validation and testing results of these CNNs. Our results outperform the baseline results, reported in [14], of 0.312 and 0.438 CCCs for arousal and valence, respectively. Additionally, they are comparable to other results from the literature (see Section 2.2).

**Figure 6:**
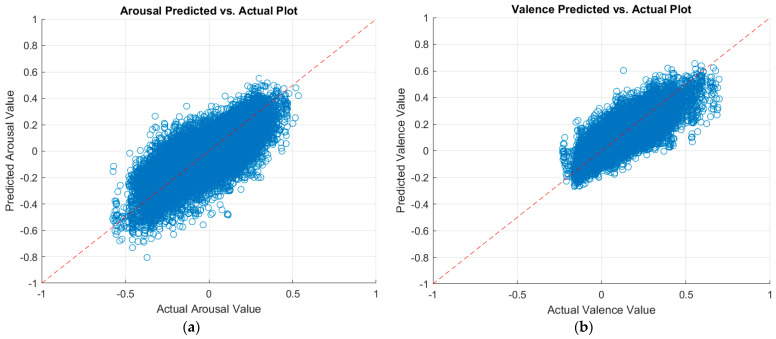
Plots of the predicted versus actual values of (**a**) arousal and (**b**) valence as per the MobileNet-v2 model.

We further experimented with the training parameter of the number of epochs. We first increased the number of epochs by another 30 epochs to a total of 60 epochs. Then, we added 90 more epochs for a total of 150 training epochs. Increasing the number of epochs leads to a longer training period; hence, more learning. Thus, a higher number of epochs can potentially enhance the prediction performance of the CNNs. Table 9 also summarizes the results of increasing the number of training epochs. The table proves that the higher the number of training epochs, the better the prediction performance. Finally, the MobileNet-v2 CNN achieved better performance over the ResNet-18 CNN. By increasing the number of epochs to 150, the MobileNet-v2 CNN achieved a testing RMSE, PCC, and CCC of 0.1220, 0.7838, and 0.7770 at arousal predictions, respectively. For valence predictions, it achieved a testing RMSE, PCC, and CCC of 0.0823, 0.7789, and 0.7715, respectively. To the best of our knowledge, these results are better than the literature. More performance enhancement is expected if the number of epochs is increased. However, we chose to stop at 150 epochs due to the time- and power-consuming process of training CNNs. Figure 6 displays a plot of the predicted arousal and valence values against the actual values, as per the best model.

### 4.3. Multimodal Fusion Results

**Figure 7:**
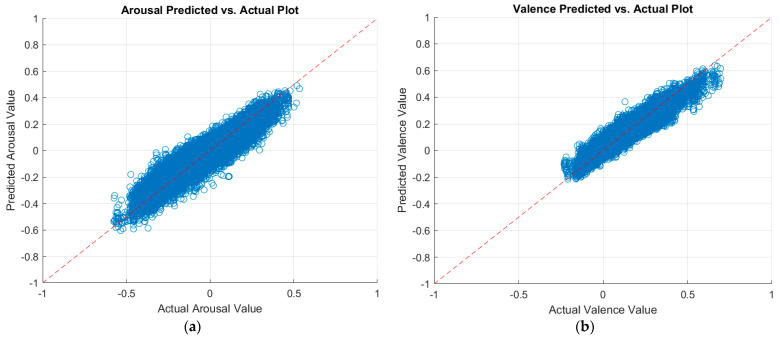
Plots of the predicted versus actual values of (**a**) arousal and (**b**) valence after multimodal decision fusion.

As described in Section 3.3.4, we applied decision fusion across the two data modalities (physiological and visual) to fuse the best predictions from the best regressor and CNN. As such, we fused the predictions of the optimized ensemble regressor for the physiological data with the predictions of the MobileNet-v2 CNN for the visual data. Table 10 shows our results after decision fusion. As can be seen, our decision fusion mechanism outperformed the results from the literature. Figure 7 displays a plot of the predicted arousal and valence values against the actual values after multimodal decision fusion.

## 5. Conclusions

In conclusion, we performed continuous emotional predictions of the arousal and valence dimensions/measures using EDA and ECG recordings and their features, as well as video recordings extracted from the RECOLA dataset. The EDA and ECG signals were processed, accompanied with pre-extracted features, and accordingly labelled with their corresponding arousal or valence annotations. Multiple regressors were trained, validated, and tested to predict arousal and valence values. We explored various preprocessing steps to study their effects on the prediction performance. The replacement of missing values and feature standardization improved the prediction performance. We also applied a feature selection mechanism which slightly improved our results on physiological data. For physiological data, the best performance was achieved by optimizable ensemble regression. To the best of our knowledge, this model outperformed previously published AR studies. This evidence points towards the use of optimizable ensemble regression for physiological data in the following steps of this project.

The video recordings were processed to extract their frames. We then applied a face detection algorithm to the extracted video frames, which were labelled with their corresponding arousal or valence annotations. For images where the algorithm failed to detect a face, manual work was carried out, which can be a time-consuming process and is sometimes prone to error. Pretrained CNNs, such as ResNet-18 and MobileNet-v2, were customized and used to predict the arousal and valence measures. MobileNet-v2 outperformed ResNet-18. Therefore, the MobileNet-v2 CNN will be retained for predicting affective states using visual data in the remainder of this project. Finally, we applied multimodal decision fusion to fuse the predictions of the optimizable ensemble on the physiological data with the predictions of MobileNet-v2 on the visual data. Our multimodal fusion results outperformed results from the literature. 

One limitation of this study is the lack of real data in the context of our specific VR application. At this stage of our work, we used the RECOLA dataset as a proof of concept. In future work, we will explore additional data modalities, additional multimodal fusion techniques, and evaluate the impact of their use in the context of a VR system. We will then apply our findings and validate our approach on real data, collected in our laboratory. Future studies could explore the use of additional sensors to not only predict affective states, but also measure cognitive effort during VR interventions, enabling the development of more personalized and effective treatments for individuals with cognitive impairments.

## Figures and Tables

**Table 2:** Physiological data breakdown.

Training Samples	Testing Samples	Total
106,201	26,550	132,751

**Table 3:** Visual data breakdown.

Parameters	Original	80–20% Split
Training Frames	106,201	84,960
Validation Frames	N/A	21,241
Testing Frames	26,550	26,550
Total	132,751	132,751

N/A (not applicable).

**Table 4:** Summary of classical machine learning data.

Dataset	Samples	EDA	ECG	Final Dimensions
Training	106,201	1 EDA reading + 62 features	1 ECG reading + 54 features	106,201 × 118
Validation	5-fold cross-validation on training data			
Testing	26,550	1 EDA reading + 62 features	1 ECG reading + 54 features	26,550 × 118

**Table 5:** BiLSTM Training Parameters.

Parameters and Options	Amount/Value
Original Sequences	132,751
Training Sequences	84,960
Validation Sequences	21,241
Testing Sequences	26,550
Sequence Length	40 ms/samples
Learning Rate	0.0001
Minimum Batch Size	9
Number of Epochs	30
Iterations per Epoch	84,960/9 = 9440
Validation Frequency	9440/2 = 4720
Hidden Units	125 × 2 = 250
Optimizer/Learner	SGDM

**Table 6:** CNN training parameters.

Parameters and Options	Original	80–20% Split
Training Images	106,201	84,960
Validation Images	N/A	21,241
Testing Images	26,550	26,550
Learning Rate	0.0001	
Minimum Batch Size	9	
Number of Epochs	30	
Iterations per Epoch	84,960/9 = 9440	
Validation Frequency	9440/2 = 4720	
Optimizer/Learner	SGDM	

N/A (not applicable).

**Table 7:** Classical regression results after feature standardization and selection.

Prediction	Regressor	Acceptable Outliers	Selected Features	Validation RMSE	Testing RMSE, PCC, CCC
Arousal	Fine Tree	100%	118	0.046975	0.0420, 0.9751, 0.9751
	Medium Tree	100%	118	0.055288	0.0494, 0.9653, 0.9651
	Coarse Tree	100%	118	0.076615	0.0697, 0.9296, 0.9279
	Optimizable Tree	100%	118	0.045176	0.0396, 0.9780, 0.9779
	Boosted Trees	100%	118	0.143590	0.1440, 0.6821, 0.5237
	Bagged Trees	100%	118	0.026890	0.0221, 0.9948, 0.9927
	Optimizable Ensemble	100%	118	0.021043	0.0170, 0.9967, 0.9958
	**Optimizable Ensemble**	**5%**	**50**	**0.020137**	**0.0168, 0.9965, 0.9959**
	Optimizable Ensemble	15%	103	0.054835	0.0533, 0.9597, 0.9574
Valence	Fine Tree	100%	118	0.026728	0.0237, 0.9830, 0.9830
	Medium Tree	100%	118	0.031906	0.0280, 0.9761, 0.9760
	Coarse Tree	100%	118	0.046718	0.0422, 0.9450, 0.9443
	Optimizable Tree	100%	118	0.025805	0.0230, 0.9840, 0.9840
	Boosted Trees	100%	118	0.099252	0.0987, 0.6784, 0.5164
	Bagged Trees	100%	118	0.016891	0.0137, 0.9958, 0.9940
	Optimizable Ensemble	100%	118	0.012907	0.0106, 0.9971, 0.9965
	Optimizable Ensemble	5%	50	0.012644	0.0099, 0.9976, 0.9969
	**Optimizable Ensemble**	**15%**	**103**	**0.010682**	**0.0083, 0.9985, 0.9978**

The rows corresponding to the highest prediction performances are displayed in bold font.

**Table 8:** BiLSTM regression results.

Prediction	Validation RMSE	Testing RMSE, PCC, CCC
Arousal	0.18461	0.1832, 0.2515, 0.1089
Valence	0.12774	0.1268, 0.1773, 0.0567

**Table 9:** CNN regression results.

CNN	Prediction	Number of Epochs	Validation RMSE	Testing RMSE, PCC, CCC
ResNet-18	Arousal	30	0.15281	0.1524, 0.5952, 0.5446
		60	0.14386	0.1443, 0.6844, 0.6353
		150	0.12683	0.1284, 0.7605, 0.7336
	Valence	30	0.11651	0.1167, 0.6720, 0.5571
MobileNet-v2	Arousal	30	0.14565	0.1458, 0.6452, 0.6192
		60	0.13653	0.1367, 0.7113, 0.7031
		**150**	**0.12178**	**0.1220, 0.7838, 0.7770**
	Valence	30	0.09890	0.0979, 0.6576, 0.6315
		**150**	**0.08309**	**0.0823, 0.7789, 0.7715**

The rows corresponding to the highest prediction performances are displayed in bold font.

**Table 10:** Decision fusion results.

Prediction	Fused Models	Testing RMSE, PCC, CCC
Arousal	Optimizable Ensemble and MobileNet	0.0640, 0.9435, 0.9363
Valence		0.0431, 0.9454, 0.9364

## Data Availability

No new data were created. Data was obtained from the RECOLA team and are available, upon request at https://diuf.unifr.ch/main/diva/recola/download.html.
